# Everyday life situations in which mothers experience difficulty stimulating healthy energy balance–related behavior in their school-age children: a focus group study

**DOI:** 10.1186/s12889-019-6826-x

**Published:** 2019-06-06

**Authors:** Emilie L. M. Ruiter, Gerdine A. J. Fransen, Gerard R. M. Molleman, Michelle J. H. M. Hoeijmakers, Koos van der Velden, Rutger C. M. E. Engels

**Affiliations:** 1 Academic Collaborative Center AMPHI, Integrated Health Policy, Department of Primary and Community Care, ELG 117, Radboud University Medical center, P.O. Box 9101, 6500 HB Nijmegen, the Netherlands; 2 Erasmus University Rotterdam, P.O. Box 1738, 3000 DR Rotterdam, the Netherlands

**Keywords:** Focus group, Mothers, Deprived neighborhoods, Parenting, Healthy energy balance–related behaviors, Difficult everyday life situations, Overweight prevention

## Abstract

**Background:**

Parental support is an important element in overweight prevention programs for children. The purpose of this study was to examine everyday life situations in which mothers encounter difficulties encouraging healthy energy balance–related behavior in their school-age children.

**Methods:**

We formed four focus groups containing 6–9 participants each. The participants were mothers of Dutch, Turkish, or Moroccan descent with a child 8–13 years of age. All focus group sessions were recorded, transcribed, and coded. Content was analyzed conventionally using ATLAS.ti 6.

**Results:**

Twenty-seven difficult everyday life situations were identified in 14 settings. The five most frequently reported situations were a daily struggle regarding eating vegetables, eating breakfast on time before going to school, eating candy and snacks between meals, and spending excessive time watching television and using the computer. A perceived loss of parental control, the inability to establish rules and the failure to consistently enforce those rules were the most commonly cited reasons for why the mothers experience these situations as being difficult.

**Conclusions:**

We identified five difficult everyday life situations related to healthy energy balance-related behavior. These five difficult situations were used as the input for developing a web-based parenting program designed to prevent children from becoming overweight. We reasoned that if we use these situations and the underlying reasons, many parents would recognize these situations and are willing to learn how to deal with them and complete the e-learning.

**Electronic supplementary material:**

The online version of this article (10.1186/s12889-019-6826-x) contains supplementary material, which is available to authorized users.

## Background

A high number of children engage in unhealthy energy balance–related behaviors (EBRBs), including excessive television watching and computer use, and low consumption of fruits and/or vegetables [1–4]. Over the long term, these behaviors cause a chronical positive net energy balance in the child, which can result in the child becoming overweight. Childhood weight problems are a major public health concern in Western countries [5, 6]. In addition, being overweight is more prevalent among children in families with low socio-economic status (SES) and families of Turkish and Moroccan descent [7, 8]. Preventing the development of overweight is important due to the high complexity of treating the condition [9], an increased likelihood of being overweight or obese in adulthood [10], and the associated detrimental health and social consequences. Negative health consequences can include developing hypertension, atherosclerosis, type 2 diabetes mellitus, and/or various forms of cancer [5]; in addition, psychosocial consequences can include depression-like symptoms [11, 12]. Together, these consequences can severely decrease health-related quality of life, contributing to rising healthcare costs [13], and even leading to premature mortality [12].

Parents can clearly influence their child’s development of healthy dietary and physical activity behaviors and are important role models both in terms of promoting these healthy behaviors in the child’s micro-environment and in terms of dealing with numerous environmental obesogenic factors [14, 15]. Published reviews increasingly emphasize the impact of parenting on preventing childhood overweight and obesity [16–21]. In addition to regular physical activity and a healthy diet, parenting styles and practices are key components of interventions designed to prevent overweight in children, and incorporating the parenting component within these interventions can greatly increase their effectiveness [17, 18, 22, 23]. The review studies by Snoek (2010) and Waters (2011) revealed that parents should be involved in interventions for the prevention of overweight [16, 23]. Parents should, for example, be supported in the following roles: *i*) helping facilitate a healthy lifestyle, *ii*) using specific EBRB parenting practices, and *iii*) learning general parenting practices [23]. However, to date, most Dutch and international obesity prevention programs have paid limited attention to parenting aspects [23–29].

According to several Dutch healthcare practitioners and policy-makers, motivating and involving parents to participate in interventions, in particular immigrant parents and parents with a low socioeconomic status (SES) is both difficult and problematic [30]. To incorporate parental involvement and support, and the parenting component (e.g. the role model, facilitator of healthy EBRB’s, and applying EBRB rules) into existing overweight prevention programs in the Netherlands, we developed a web-based Dutch parenting program (also known as an e-learning program) for parents of children 8–13 years of age. We chose parents of children 8–13 years of age, because we selected the already existing school-based overweight prevention intervention, entitled “Scoring for Health” to which we will add the e-learning. Scoring for Health is offered on a large scale to primary schools in low-SES neighborhoods for children 8–13 years of age. The e-learning program’s effectiveness will be tested in an upcoming cluster randomized controlled trial [31]. Importantly, parents will be able complete the e-learning program in their homes, at a time that suits them. The e-learning program will teach parents how to encourage and support their child’s decision to develop and maintain healthy EBRBs, as well as how to handle everyday life conflict situations that can jeopardize healthy EBRBs.

Moreover, it is important to ensure that overweight prevention programs fit the lifestyle and needs of the parents and children. Previous studies have investigated the challenges that parents face when attempting to provide their children with healthy EBRBs, parents’ perceptions regarding healthy behaviors, and parents’ opinions regarding obesity prevention programs [32–36]. However, little is known regarding the specific everyday life situations in which parents experience difficulties; moreover, the underlying reasons for why parents encounter these difficulties are poorly understood. We reasoned that if we use these specific situations and the underlying reasons when creating our e-learning program (based on the theoretical insights from Parent Effectiveness Training and Parent-Management Training-Oregon Model [37, 38]), many parents would recognize these situations and would be willing to learn parenting skills that teach them how to deal with difficult everyday life situations. Thus, many parents would feel more compelled to complete the e-learning program. Studies have shown that when a message is personally relevant to someone, that individual will encode and internalize the message more efficiently [39]. Thereby this is increasing the likelihood of achieving a more positive attitude and a more favorable outcome; importantly, this change in attitude is often associated with a change in behavior [40, 41].

Therefore, as a first step towards developing this e-learning program and to get more insight into determinants of everyday EBRB parenting, we conducted focus groups with mothers who live in low-SES neighborhoods in the Netherlands. We chose for parents who live in low-SES neighborhoods, because low-SES parents are difficult to reach with interventions [30], and also the group where childhood overweight is more prevalent [8]. The aims of this study were to *i*) explore and identify everyday life situations in which mothers experience difficulty stimulating healthy EBRBs in their school-age child, and *ii*) identify the reasons why mothers encounter these difficulties.

## Methods

### Study design

We chose to study focus groups rather than conducting interviews and/or questionnaires, as focus groups are particularly valuable for exploring the experiences and issues that are important to the participants, and it allows the participants to provide this information using their own words and phrases [42]. A focus group encourages interaction between participants, which facilitates a rich discussion, and group discussion can encourage contributions from people that may normally not respond [42]. In addition, focus groups are an appropriate means to approaching low-SES groups, because the interaction that focus groups bring, allows groups of peers to express their perspective. Having the security of being among others who share many of their feelings and experiences, the participants possess a basis for sharing their views [43]. Further, focus groups do not discriminate against people who cannot read or write and they can encourage participation from people reluctant to be interviewed on their own or who feel they have nothing to say [42].

Because the prevalence of overweight children varies with ethnicity, we selected ethnically heterogeneous groups comprised of mothers of Dutch, Turkish, and Moroccan descent. On the other hand, we attempted to make the groups as homogeneous as possible with respect to other factors, including the children’s age, the parents’ gender (all parents were female) and neighborhood (low-SES, based on lower levels of education and lower incomes), thereby facilitating communication and ensuring that the majority of participants share their experiences with their peers.

The Medical Review Ethics Committee of the Region Arnhem-Nijmegen, the Netherlands approved this study (Reg. nr.: 2012/145).

### Participants

In the spring of 2012, mothers of children 8–13 years of age were invited to meet as a group with other mothers to discuss their opinions and experiences regarding encouraging healthy EBRBs among their children. A purposeful sample of mothers was recruited. The inclusion criteria for participation were as follows: mothers who live with a child 8–13 years of age in a low-SES neighborhood (because the e-learning is intended for parents live with a child 8–13 years of age in a low-SES neighborhood); families of Dutch, Turkish, or Moroccan descent; and an understanding of the Dutch language at the speaking level. We included only mothers, because they are often the primary caretakers. Moreover, we wanted to create a safe focus group for the immigrant mothers in which they all dare to talk [44]. That is why we have chosen only mothers instead of mothers and fathers together. Each focus group consisted of a combination of Dutch, Turkish, and Moroccan mothers.

### Recruitment

We contacted six primary schools by telephone, each of which was located in another low-SES neighborhood in Nijmegen, the Netherlands. All six primary school principals gave permission to recruit mothers at their school. Key informants, which were female volunteers who routinely organize various parent activities, were asked to help recruit mothers face-to-face who fulfilled the inclusion criteria. Using a purposeful sampling strategy, these key informants directly contacted mothers who were likely to participate and arranged a convenient date and time for the mothers. All contacted mothers agreed to participate. The key informants at four schools successfully recruited an ethnically heterogeneous group of mothers. All recruited mothers showed up for the focus group conversation. The informants at the two remaining schools failed to recruit an ethnically heterogeneous group, as these schools contained no Dutch-speaking Turkish or Moroccan mothers; therefore, no focus groups were performed at these two schools.

### Focus groups

The four focus group meetings were held in a multifunctional room at their school; these locations were chosen because various child and parent activities are held there, and the rooms were therefore familiar to the participants. All participating mothers provided written informed consent and gave permission for making an audio recording of the meeting. The participants also completed a brief socio-demographic questionnaire. Each focus group session lasted approximately 2 hours.

The focus group meetings were guided by a trained moderator (author E.R.) with the support of a trained assistant (author M.H.). The moderator was female and worked as a youth health care doctor (MD) and PhD-student. She followed a certified training [45] on moderating focus groups, where she tested the semi-structured interview guide (See Additional file 1). Prior to the study, there was no relationship with the participants. The moderator facilitated the discussion, asked questions, and probed for more information to elucidate the participants’ comments (e.g. ‘Can you give an example of how this is done in your home?’ or ‘Why is that situation so difficult?’). The assistant took detailed notes and tracked the individual contributions of each participant. The moderator used a semi-structured interview guide, which was based on the research questions. The mean questions were “Which factors are promoting or hindering you as a parent in promoting healthy eating and physical activity habits in your child?” and “In which everyday life situations do you experience difficulties?” The questions were designed to be open-ended. To increase the credibility, member checking was conducted between each focus group question and at the end of each focus group to make certain that the moderator accurately understood the answer provided by the participants [46, 47]. During a brief “warm-up” session, the moderator asked mothers to list in writing—as quickly as possible—all of the words that came to mind when they first thought about diet and their child; in the second part of the meeting, they were asked to list the words that came mind when thinking about physical activity and their child. After these brief warm-up sessions, the moderator used open-ended questions to start the discussion and then focused on elucidating the mothers’ responses. Thereafter, the moderator focused specifically on asking which everyday life situations mothers experience difficulty when encouraging their children to maintain a healthy EBRB. Finally, the questions from the moderator focused on elucidating the comments in order to better understand why some mothers find it challenging to encourage healthy EBRB in their children. All focus group sessions were recorded, and transcribed verbatim. The participants were rendered anonymous by assigning each participant a numeric code. Transcripts were not returned to participants for comment and/or correction.

### Data analysis

We performed a conventional qualitative content analysis in order to inductively derive quotations and subsequent themes from the data [48]. The data were analyzed using the ATLAS.ti 6 software package. Quotations were chosen based on the unit of analysis, which was defined as: all text passages containing any information about daily routine pursuits or situations regarding diet, physical activity, or sedentary behavior that were considered difficult by the mothers. A difficult everyday life situation was linked to a specific activity (e.g., watching television) and—where possible—to a setting (e.g., eating fruit at school). All comments regarding the reasons why mothers reported difficulties were categorized as child-related factors (e.g., preference for a certain food), parental factors (e.g., inconsistent parental practices), environmental factors (e.g., school, friends, etc.), or “other”.

To increase the dependability [46, 47], two researchers (authors E.R. and M.H.) independently highlighted quotations in the transcripts from the first two focus groups and assigned codes to all quotations. Thereafter, the code lists were discussed, and consensus was reached regarding the final codes. To obtain a code tree, the codes were independently grouped into categories and themes by two researchers (authors E.R. and M.H.), and the code tree was discussed in order to reach consensus. The other two focus groups were coded together, and the code tree was adjusted accordingly. To increase reliability, the code tree was reviewed, discussed, and confirmed by an independent member of the research team (author G.F.). Participants were not asked to provide feedback on the findings.

Using an iterative approach, the researchers reviewed the transcripts in order to reflect upon each focus group meeting prior to conducting the next focus group meeting, thereby allowing newly identified codes and themes to be incorporated into subsequent sessions. After the four focus group meetings, recruitment was stopped because data saturation had been reached. This point was defined as no new themes emerged from the fourth focus group.

A Dutch-to-English translator at the Radboud University Nijmegen translated all of the quotations used in this publication.

## Results

### Participant characteristics

A total of 28 mothers participated in the four focus groups, including the four key informants and one volunteer in the multifunctional room at one school. Each focus group consisted of 6–9 mothers, with a total of 13 Dutch, 1 German, 8 Turkish, and 6 Moroccan mothers. Each of the four focus groups contained a mixture of mothers from Dutch, Turkish and Moroccan descent. Five ethnic Dutch mothers were married to a man of Turkish (*n* = 4) or Moroccan (*n* = 1) descent. The demographics of the participating mothers are summarized in Table 1. With respect to education, 71.4% of all participants completed only primary school or high school, and nearly 40% of all participants were unemployed at the time of their respective focus group meeting.

**Table 1:** Demographics of the mothers who participated in the focus groups (*n* = 28)

	*n*	*%*
Age (years)		
20–30	2	7.1
31–40	12	42.9
41–50	12	42.9
>51	2	7.1
Race/Ethnicity		
Dutch	13	46.4
Turkish	8	28.6
Moroccan	6	21.4
Other	1	3.6
Highest level of education achieved		
Primary school	5	17.9
High school	15	53.6
Secondary vocational education	7	25.0
Higher vocational education	1	3.6
Employment status at the time of the focus group		
Full-time paid employment	4	14.3
Part-time paid employment	11	39.3
Volunteer work	2	7.1
Unemployed	11	39.3
Marital status		
Married	17	60.7
Divorced	6	21.4
Cohabitation	3	10.7
Single	2	7.1
Number of children		
1 child	3	10.7
2–3 children	20	71.4
4–6 children	5	17.9
Perception of their child’s weight status (*n* = 37 children)		
Underweight	3	8.1
Normal weight	29	78.4
Overweight	4	10.8
Incomplete data set	1	2.7

The total number of children per mother ranged from one to six. Among the 23 recruited mothers, 15 had one child, 7 had two children, and 1 had three children 8–13 years of age. The four key informants and the volunteer failed to meet this age-based criterion; specifically, three of these participants had one 3-year-old child, one had a 5-year-old child, and one a 22-year old child; the other two participants each had a 15-year-old child. The primary conversation and qualitative analysis focused on the children who were 8–13 years of age.

### Difficult everyday life situations

The difficult everyday life situations that were mentioned in the focus groups are summarized in Table 2. Data analysis identified 14 major activities and/or settings in which mothers encountered 27 difficult everyday life situations with respect to encouraging healthy EBRB in their children. With respect to encouraging a healthy diet in their children, the following activities/settings emerged from the analysis: (1) just before eating dinner, (2) at the dinner table, (3) just before eating breakfast, (4) at the breakfast table, (5) eating candy and/or snacks at home, (6) eating candy and/or snacks at school, (7) eating candy and/or snacks elsewhere, and (8) eating fruit at school.

**Table 2:** Difficult daily situations for mothers in encouraging healthy energy balance–related behavior in their school-aged child

Setting and/or activity	Difficult everyday life situation Mothers find it difficult when/that their…
Having dinner	
(1) Just before dinner	Children complain about the kinds of foods the parents are cooking
	Children do not want to sit at the table when told
(2) At the dinner table	Children do not want to eat their vegetables
	Children’s preferences for vegetables change over time
	Children eat extremely slowly
	Children do not eat enough
Having breakfast	
(3) Just before breakfast	Children have trouble getting out of bed
	Children do not want to sit at the table when told
(4) At the breakfast table	Children do not eat well in the morning
	Children eat slowly when there is not enough time
Eating candy and snacks	
(5) At home	Children constantly want to eat candy/snacks
	Children eat candy/snacks when the parents are not around
(6) At school	Children are given unhealthy treats
(7) Elsewhere	Children buy candy and snacks themselves
	Children are given candy and snacks by their friends
	Children are given candy and snacks by their grandparents or other family members
Eating fruit	
(8) At school	Children do not always eat their fruit or do not eat any fruit at all
	Children want the same unhealthy snacks as their classmates, rather than fruit
Physical activity	
(9) Playing sports	Children do not want to participate in sports and are physically lazy
	Children are not able to choose a sport
(10) Playing outside	Children do not feel like playing outside
	Children play outside by themselves
Sedentary behavior	
(11) Computer use at home	Children spend an excessive amount of time at the computer
	Children do not want to turn off their computer game when they are told to do so
(12) Computer use elsewhere	Children spend an excessive amount of time playing on a computer at their friend’s house
(13) Watching television at home	Children spend an excessive amount of time watching television
(14) Watching television elsewhere	Children watch an excessive amount of television at their friend’s house

The remaining six settings were considered less difficult and were discussed less frequently; these settings were associated with encouraging physical activity and discouraging sedentary behavior, and included: (9) playing sports, (10) playing outside, (11) computer use at home, (12) computer use elsewhere, (13) watching television at home, and (14) watching television elsewhere. Notable findings are highlighted below per setting.

#### Dinner: just before dinner and at the dinner table

The mothers identified four difficult situations that arise near dinner time. (1) Mothers noted that they sometimes become frustrated before dinner is served because of the effort it takes to get the children to the dinner table and because children preferred to remain outside and play or were busy on the computer. (2) Mothers complained that their children would start whining about the food while it is being prepared. Mothers also noted that it can be very discouraging when the children do not want to eat the healthy meal that they prepared, feeling as though it was a waste of effort and time.

(3) The most frequently cited situation in each focus group (and within each ethnic group) was the struggle or conflict surrounding eating at the dinner table. The primary conflict was due to their children’s refusal to eat vegetables, which can lead to frustration and anger from both the parents and the child. One mother (Dutch/Turkish, 35 years of age) said, “*That discussion.. My child might say, ‘If I eat the mushrooms, can I leave the peppers on my plate?’ …that everlasting struggle while eating… I get so tired of that”.* (4) Some mothers mentioned that they find it difficult that their children were extremely slow eaters and/or they are concerned that their children do not eat enough and therefore must be forced to eat. As one Dutch mother (42 years of age) noted, *“She was supposed to eat her vegetables, and I was sitting at the table and shouted, ‘And now you eat!’.”*

#### Breakfast: just before breakfast and at the breakfast table

Although the majority of mothers reported that their children eat breakfast each morning the mothers experienced similar difficulties as with dinner. Several mothers complained that the children are slow to get out of bed leaving little time for breakfast on weekdays. As one Turkish mother (37 years of age) said, *“During the week we always have to hurry… the children don’t get out of bed easily… at ten minutes to eight they still aren’t up, and then we don’t have any time to sit down and have breakfast together.”*

#### Eating candy and snacks at home, at school, and elsewhere

The children were reported to prefer to eat candy and snacks all day, as noted by one Turkish mother: *“They want to eat it twenty-four hours a day…”* On the other hand, most mothers were cognizant of the fact that children often eat candy and snacks when the parents are not in the vicinity or are not watching the child.

The most frequently cited issue was the mother’s perceived loss of control regarding the child’s consumption of candy and snacks when the child is not at home. Children often buy unhealthy foods using their own money or they get these foods from friends, grandparents, or other family members, who then become negative role models with respect to diet. The mothers felt they had no control over this practice, as noted by a 29-year-old Dutch mother: *“I’m constantly saying ‘no’. But his grandparents are always saying, ‘Oh go ahead, take it’.”* At school, the children often get unhealthy foods from other children, as noted by a 39-year-old Dutch mother: “*These days the kids get big jars of candy as treats. Truly, my daughter comes home with these big jars full of candy; it’s ridiculous.”*

#### Eating fruit at school

Eating fruit at home was generally not reported to be a problem; all of the mothers indicated that their children like eating fruit and eat fruit every day. However, eating fruit at school was reported by some mothers as being problematic. For example, a 49-year-old German mother said “*…the children don’t have the opportunity to eat fruit. They get a fifteen-minute break, and in that time they have to eat their fruit, drink, and play outside.”* On the other hand, some children observe other children eating unhealthy snacks or cookies during the school break and therefore do not want to eat fruit at school. This presents a challenge to the mothers, as they want their children to eat fruit at school.

#### Physical activity: playing sports and playing outside

A couple of mothers noted that it can be difficult to engage their children in sports. For example, a 37-year-old Dutch/Turkish mother noted, “*He is very stiff. I ask him, ‘Do you want to play hockey or tennis or…?’ But nothing really interests him.”* However, most of the mothers reported that encouraging their child to engage in sports is generally not difficult. As one Turkish mother said, *“What kid would not like sports anyway?”* A Moroccan mother mentioned, *“That really goes without saying.”*

Most of the mothers noted that their children like to play outside and play outside regularly. On the other hand, mothers often find it difficult to accept the lack of control when their child plays outdoors alone; safety on the street was one of the issues mentioned. This can influence the time of day and duration of when the child is allowed to play outside; a 40-year-old Turkish mother stated, *“… with playing outside I’m a little afraid, since we live in a bad neighborhood; so I have very strict rules. It is difficult though, so I try to keep him home more often because he’s not allowed to go outside in the evening.”*

#### Sedentary behavior: computer use and watching television at home and elsewhere

In all four focus groups, the mothers agreed that attempting to discourage sedentary behavior among their child was more difficult than encouraging their child to be physically active. Most children prefer watching television and sitting behind the computer, sometimes for several hours; however, computer use was considered more problematic than watching television. If their children are not watching television or playing video games at home, they are doing it at their friend’s place. A 35-year-old Moroccan mother said, “*My youngest enjoys playing computer games and watching television. Yesterday the weather was really nice so I tried to get him outside by forbidding him to stay indoors… …So when I finally got him out the door he went to his neighbor, his friend, and they went indoors and watched television and played computer games for hours!”*

### Reasons why mothers experience difficulty encouraging healthy EBRBs among their children

Aside from the difficult everyday life situations described above, mothers also discussed the reasons why they experience problems when attempting to encourage a healthy diet and physical activity while discouraging sedentary behavior. A total of 11 reasons were discussed, and these are divided into parental, child-related, and environmental factors (Table 3). The more notable findings are discussed below.

**Table 3:** Reasons why mothers in low-SES neighborhoods experience difficulty in encouraging healthy energy balance–related child behavior

	Example quotes by mothers
(1) Parental factors	
Parenting difficulties	
(a) Not always as strict and consistent	*“…at a certain point you don’t feel like struggling anymore.…” (Dutch, 49 yrs)*
	*“When you nag too much you feel like you are a bad mother.” (Dutch, 39 yrs)* “*The problem with (my child) is that he snacks too much. I find that very difficult. He can’t just eat one single candy; he needs to eat way more than just one.” (Dutch, 29 yrs)*
(b) Remain calm is considered difficult	*“…sometimes when you got really angry, you regret raising your voice that much. However, sometimes yelling is required to make them see that you are serious.” (Dutch/Moroccan, 30 yrs)*
(c) Lack of parental rules	*“My son prefers to sit in front of the TV all day. I have to admit that it’s nice from time to time since my son is really hyperactive. So that’s some quiet time for him.” (Dutch/Turkish, 35 yrs)*
(d) Negative food role modeling	*“…actually I’m a bad role model since I don’t have breakfast myself.” (German, 49 yrs)*
	*“I myself am love sweet stuff. I find it very difficult to tell my kids that they can’t have candy.” (Turkish, 40 yrs)*
(e) Father and mother have different parenting rules	“If their father is home, the children eat fewer vegetables. I still say that they should eat some, but their father says “just leave (the kids) alone”....” *(Moroccan, 42 yrs)*
(g) Loss of parental control	*“I’m not comfortable when he’s outside... I’d rather have him at home.” (Dutch/Moroccan, 30 yrs)*
	*“... I know others give my child treats. They aren’t supposed to. But you cannot be in two places at once as a parent.” (Moroccan, 43 yrs)*
(h) Mother’s emotional status	*“You always do your best, but still you know you are doing some things wrong. However, these are those moments, it all depends on how your kids are feeling, but also how you yourself are feeling.” (Dutch, 42 yrs)*
Lack of knowledge & misconceptions	*“They need it. I think that… they lose energy so quickly that they need it again. I also experience that myself, sometimes you are like ‘oh, I just need something sweet’.” (Turkish, 37 yrs)*
	*“When they are young you can encourage them, but when they get older (it is more difficult).” (Moroccan, 43 yrs)* *“…* as long as my child is active, eating candy and/or snacks is not much of a problem.” (Dutch, 39 yrs)
(2) Child factors	
(a) Child’s behavior	*“…I know I have to stay patient, I know what I have to do, but they resist.” (Turkish, 37 yrs)*
	*“…when he is really hyperactive I notice that I, at a certain point, get a bit exhausted.” (Dutch/Turkish, 35 yrs)*
(b) Child’s preferences	*“…with sandwich spreads I have difficulties, especially with the youngest; she always wants chocolate spreads or sprinkles. She absolutely doesn’t want any cheese; she doesn’t like cheese.” (Turkish, 40 yrs)*
	*“They don’t like much food, except pancakes and fries. ‘Picky’… yes that’s the correct word!” (Dutch/Turkish, 35 yrs)*
(3) Environmental factors	*“The healthy treats that they give, that’s only every now and then. The teachers aren’t strict enough, which I find a pity!” (Dutch, 47 yrs)* *“… with playing outside I’m a little afraid, since we live in a bad neighborhood; so I have very strict rules. It is difficult though, so I try to keep him home more often ...”. (Turkish, 40 yrs)*

#### Parental factors

##### Parenting difficulties

The act of parenting itself was considered to be challenging; a 39-year-old Dutch mother noted, *“You know... parenting without experiencing conflicts is just not possible.”* In particular, despite having established rules, mothers mentioned that they are not always as strict and consistent as they would like, and that they often give in too easily. Many mothers mentioned that the ability to remain calm was difficult; and also mentioned that raising their voice and even yelling is often unavoidable and even necessary in order to show that the child that she is serious. A lack of rules was also mentioned as a problem. In contrast with clear food-specific rules, many of the mothers indicated they have far fewer rules regarding physical activity, and very few mothers mentioned they have rules regarding computer use. Although most mothers do not want their children to spend more than 1 hour on the computer, this limit is often exceeded because the mothers do no paid attention to the child’s computer use. Most mothers mentioned they do not have specific rules regarding television viewing; they merely decide when the television should be turned off. Although all of the mothers indicated that they serve as a role model for their child, they do not always behave as such. Some mothers do not eat breakfast themselves, yet attempt to encourage their child to eat breakfast. Furthermore, mothers indicated they like eating candy, snacks, and fast food from time to time, but do not want their child eating these foods. With respect to dinner, some mothers only cook foods that they like, automatically restricting the child’s food choices. With the exception of breakfast, negative food-related role modeling was not generally considered as a problem by the mothers. Some mothers mentioned that they find it difficult when the father and mother have different parenting styles. In addition, almost all mothers indicated they find it difficult to perceive a loss of parental control regarding their children as their children grow, and when they spend time with others (e.g., grandparents and friends) who undermine their rules. Lastly, the mothers indicated that their own emotional status can serve as a perceived barrier against encouraging healthy behavior in their children. For example, some divorced mothers expressed that they find it difficult to deal with the consequences of divorce and raising a child on their own.

##### Lack of parental knowledge and misconceptions

In general, the mothers’ knowledge regarding the importance of eating breakfast, eating vegetables, and physical activity was quite accurate. Nevertheless, they lacked knowledge with respect to the daily recommended consumption of vegetables and the requirements regarding physical activity. In addition, certain misconceptions emerged, for example regarding the notion that children can no longer be encouraged when they grow older. Another example is that some mothers believe that children need candy for the energy it provides. Thus, as long as a child is active, eating candy and/or snacks is not much of a problem.

#### Child-related factors

First, with regard to the **child’s behavior**, most of the mothers noted that they find it difficult to deal with children who are hyperactive, dominant, stubborn, or refuse to listen and find it more difficult to apply and enforce consequences upon children who exhibit these behaviors, often resulting in a confrontation between the parent and child. For this reason, mothers declared they often prefer to avoid certain conflicts and simply “give up”. Second, mothers mentioned they experienced problems with dealing with the **child’s preference** for sweet foods. With respect to dinner, many mothers mentioned their children were “picky eaters”. Mothers indicated that both remain a common cause of conflicts, and that they are difficult to deal with.

#### Environmental factors

Lastly, the mothers in the focus groups talked about the inconsistent food policies at their child’s school. According to the mothers, several campaigns to encourage school-age children to eat fruit and healthy treats at school were not maintained or enforced by the school. Most of the mothers mentioned they are in favor of schools adopting consistent rules and establishing a consistent policy with respect to food at school. This would then make it easier for them to establish and enforce similar rules at home.

## Discussion

Our aim was to explore the difficult everyday life situations that mothers in low-SES neighborhoods face with respect to encouraging healthy EBRBs in their school-age children. Although previous studies identified several barriers that prevent parents from providing their children with a healthy lifestyle [32–36], our results describe real-world everyday life situations that mothers perceive as challenging. In the focus groups, the mothers reported several everyday life difficulties with respect to eating dinner, eating breakfast, avoiding candy and snacks, eating fruit at school, playing sports, playing outdoors, restricting computer use, and limiting television time. In addition, this study provides important insights into the reasons why mothers encounter these difficulties.

The most frequently cited problematic and discouraging situation for the mothers was the daily struggle at the dinner table. This finding supports the results of a cross-sectional study that examined parents’ perceptions of the mealtime environment [49]. One of the mealtime challenges of 40 % of the parents was a “conflict about food” on a daily basis, and these conflicts were related to the child’s “pickiness” regarding food [49].

The mothers in our focus groups also worry about their child’s constant desire for candy and snacks, as well as the negative influence of friends and other peers on the child’s eating behavior. The mother’s feeling of “losing control” and that other individuals undermine their rules were considered to be particularly difficult. This finding is consistent with a focus group study by Hart et al., who qualitatively investigated parental barriers and benefits for providing children with a healthy diet and adequate exercise. Siblings, non-resident parents, grandparents, and the child’s friends were all viewed as potentially negative food role models by both low-SES and high-SES mothers of children 7–12 years of age [50]. Furthermore, in a large survey conducted among Dutch children 4–16 years of age and their parents, 16% of parents indicated that they found it difficult to not have control over what their child eats [51].

The feeling of losing control when the child plays outdoors was also reported in previous studies, and is often referred to as a “lack of perceived neighborhood safety”. For example, inner-city parents are considerably more anxious regarding neighborhood safety than suburban parents, and this concern is inversely correlated with the child’s level of physical activity [52]. In their review, Carver et al. concluded that road safety and “danger of strangers” are responsible for most parents’ anxieties related to the child’s safety when playing outside. However, to date, little evidence is available to suggest that this has an impact on the child’s physical activity and walking and/or biking to school [53].

Only a few mothers in our focus groups mentioned encouraging their child to be physically active as a specific problem. Moreover, the consumption of sugar-sweetened beverages (SSBs) was not mentioned at all by the mothers. However, research by the Municipal Health Service, region Nijmegen and national research found that the majority of children do not meet established physical activity recommendations, and one in four children 0–12 years of age consumes at least three SSBs per day [1, 54, 55]. For example, in 2011 and 2013, respectively 16 and 21% of children 4–12 years of age met the Dutch recommendation for physical activity [54, 55].Thus, mothers in low-SES neighborhoods might overestimate their child’s physical activity while underestimating their child’s intake of SSBs. A possible explanation for this could be that the mothers are simply not aware of their child’s activity level and SSB consumption outdoors (e.g. at school or at children’s friends’ houses). Or because mothers think the physical activity level and SSB consumption of their child is within the healthy norm and not a problem, due to their lack of knowledge regarding physical activity recommendations, SSB consumption recommendations, and health risks of high SSB consumption.

Although the mothers stated that their child’s screen time (e.g., computer use and television watching) was a problem, they noted that they did not establish clear rules regarding using the computer and—in particular— watching television. This is in line with Jordan’s findings among parents of children 6–13 years of age, that reported that only few parents had rules restricting the time children spend watching television [56] and also in line with the findings of a survey of the Municipal Health Service in the Nijmegen Region [1]. Establishing rules and limits regarding screen time can be effective at reducing the time that children spend performing screen-based activities [57]. Therefore, interventions should provide parents with information to help them establish rules regarding screen-time activities in order to reduce their children’s screen time.

One important finding is that mothers in low-SES neighborhoods indicated that they face several difficulties with respect to parenting. The inability to establish rules and the failure to consistently enforce those rules were mentioned most frequently. Moreover, the mothers indicated that they would like to see a consistent healthy food policy established in their child’s school, which would support them in their efforts to promote healthy dietary behavior in their child. According to other Dutch data, more than one-third of parents experience parenting difficulties at some time, and this percentage is even higher among parents with low SES; low-SES parents also express a greater need for parenting support [58].

Despite its benefits, this study also had limitations that warrant discussion. First, the purposeful sampling strategy does potentially not reflect the general population. The mothers were recruited using so-called “key informants”. Thus, it is possible that these mothers were highly motivated and interested in their children’s health, as they are often involved in school activities. Furthermore, the key informants also participated in the focus groups, although they did not have children 8–13 years of age. However, none of these mothers were excluded, as they contributed to the discussion and the other mothers responded to their input. The informants were mothers who are connected to the living room projects at the primary schools and trusted persons (peers) for our participants. The informants had the same cultural background as the participants, which ensured a safe, secure atmosphere during group consultations. Nevertheless, our analysis revealed that omitting the statements made by the key informants did not change the primary findings of this study. Moreover, it is possible the mothers simply gave the answers that they felt to be socially desirable. However, we made every effort to make the focus group an open, safe environment; consequently, the mothers seemed to feel comfortable, so we believe that they provided honest answers.

Because of the relatively small sample size and the use of purposeful sampling, our findings do of course not include the experiences of all mothers who live in low-SES neighborhoods within the Netherlands extensively. Yet, our findings provide an approximation of the situation experienced by mothers in low-SES neighborhoods in the region, as we included ethnically diverse mothers from four different low-SES neighborhoods throughout the city, and data saturation was achieved after four focus groups. Moreover, the views represented by fathers were not included in this current study. Studies show that the parenting styles of fathers and mothers have different effects on the EBRBs of the child [21, 59]; therefore, it would be interesting to conduct future focus groups with fathers to determine whether their opinions and problems differ from those of the mothers. Finally, additional research should investigate further parental experiences regarding parenting in general and/or whether parents of children with overweight or obesity experience different challenges.

### Implications for practice

Based on this focus group study, we identified everyday life situations in which mothers experience difficulty stimulating healthy EBRBs in their children. In addition, we found that the parenting difficulties mentioned by mothers can be subdivided into the following three negative core dimensions of parenting style as describes by Skinner [60]: i) rejection: irritability and difficulty remaining calm, ii) chaos: inconsistency, father-mother inconsistency, and a lack of parental rules, and iii) coercion: punitive measures and forcing the child to eat. In addition, we found that mothers who live in a low-SES neighborhood were easily willing to participate in the focus groups and discuss EBRB-related topics. Thus, these mothers value EBRBs and seem willing to learn how to deal with these everyday life difficulties. These results were used as input for developing our e-learning program for parents of children 8–13 years of age in order to prevent weight problems. The difficult situations and the transcripts from the focus group meetings were used for the content of the e-learning program. In this e-learning program, parents receive tools that they can use to encourage their child to develop healthy EBRBs. These tools use both general and specific parenting and conflict-management approaches [37, 38, 61]. By using the information obtained directly from the parents in our focus groups to develop our e-learning program, and by using video clips that show how difficult situations handled using both “good” and “less good” approaches, parents will likely identify with the everyday life situations described in the program. We therefore expect that parents will feel compelled to follow the program and will be more willing to learn new parenting skills in order to help them overcome these difficult situations. Nevertheless, we will perform a randomized controlled trail in order to investigate parents’ willingness to follow the e-learning program, as well as the effects of the program on parenting styles and practices and on EBRBs among children 8–13 years of age.

## Conclusions

Mothers who live in low-SES neighborhoods in the Nijmegen region in the Netherlands reported experiencing many difficulties in everyday life situations when attempting to stimulate healthy dietary behavior and discourage excessive television watching and computer use by their children. Parenting was considered to be a difficult task, and this was reported as an important reason for why mothers encounter these difficult everyday life situations. These important insights—and the transcripts from the focus group meetings—has been used as the input for developing a web-based parenting program for parents of children 8–13 years of age in order to prevent weight problems.

## Additional file

Additional file 1: The semi-structured interview guide used by the moderator. (DOCX 17 kb)

## Abbreviations

EBRB: energy balance–related behavior
SES: socio-economic status
SSBs: sugar-sweetened beverages
