# Supplementary material for: Everyday life situations in which mothers experience difficulty stimulating healthy energy balance–related behavior in their school-age children: a focus group study
Source: BMC Public Health. 2019 Jun 6;19:701. doi: 10.1186/s12889-019-6826-x (PMC6555719; doi:10.1186/s12889-019-6826-x)
Supplement: Supplementary file 1 — The semi-structured interview guide used by the moderator. (DOCX 17 kb) [file 12889_2019_6826_MOESM1_ESM.docx]

**Additional file 1. Semi-structured interview guide**

1. **Main question 1 (opening question for part 1): What comes to mind when you think about diet and your child?**

*Clarifying questions:*

- Can you explain why you thought of this?
- Can you give an example of how this is achieved in your family/home?

1. **Main question 2: Which factors either help or hinder you as a parent in terms of promoting healthy dietary behavior in your child?**

*Clarifying questions*:

- What works in a positive manner or helps stimulate your child?
- In which way can you teach or encourage your child to eat a healthier diet?
- Why does this work in stimulating your child?
- What does not work, and what do you experience as difficult?
- Why do you experience this as being difficult?
- Can you give an example of how this is achieved in your home?

1. **Main question 3 (opening question for part 2): What comes to mind when you think about physical activity and your child?**

*Clarifying questions:*

- Can you explain why you thought of this?
- Can you give an example of how this is achieved in your home?

1. **Main question 4: Which factors either help or hinder you as a parent in terms of promoting healthy physical activity behavior in your child?**

*Clarifying questions*:

- What works in a positive manner or helps stimulate your child?
- In which way can you teach or encourage your child to be more physically active?
- Why does this work in stimulating your child?
- What does not work, and what do you experience as difficult?
- Why do you experience this as being difficult?
- Can you give an example of how this is achieved in your home?

1. **Main question 5: When you think of an average day or week, in which everyday situations do you experience difficulties promoting healthy dietary and physical activity behaviors in your child?**

*Clarifying questions:*

- Why do you think these situations are so difficult?
- What factors make this situation more difficult?
- Are there any other difficult situations that we have not yet discussed?
